# Development and validation of a risk prediction model for acute kidney injury in patients with malignant obstructive jaundice: a retrospective matched cohort study

**DOI:** 10.1080/0886022X.2025.2570813

**Published:** 2025-10-13

**Authors:** Pingping Ju, Zhaoting Li, Yu Zhu, Chunbo Zou

**Affiliations:** ^a^ Department of Nephrology, The Affiliated Taizhou People’s Hospital of Nanjing Medical University, Taizhou School of Clinical Medicine, Nanjing Medical University, Jiangsu, China; ^b^ Department of Nephrology, Taicang Loujiang New City Hospital (Ruijin Hospital Taicang Branch), Jiangsu, China

**Keywords:** Acute kidney injury, malignant obstructive jaundice, prediction model, logistic regression, nomogram, propensity score matching

## Abstract

Acute kidney injury (AKI) is a common and serious complication in patients with malignant obstructive jaundice (MOJ), yet no predictive model exists for this specific population. This retrospective study included 557 hospitalized MOJ patients, with 103 developing AKI. Propensity score matching was used to control confounding, and 103 matched pairs were analyzed. Least absolute shrinkage and selection operator (LASSO) regression and multivariate logistic regression were used to develop the prediction model. Model performance was evaluated using the area under the curve (AUC), concordance index, Brier score, calibration curve, decision curve analysis, and clinical impact curve. Seven variables were included in the final model: neutrophil-to-lymphocyte ratio (NLR), C-reactive protein (CRP), uric acid (UA), total bilirubin (TBil), potassium (K), carbon dioxide combining power (CO_2_CP), and prothrombin time (PT). The model showed excellent discrimination (AUC = 0.891) and clinical usefulness, with good calibration demonstrated by the Hosmer–Lemeshow test (*p* = 0.567) and internal validation using bootstrap resampling (*B* = 1000). A nomogram was constructed for individualized risk assessment. Risk stratification based on predicted probabilities showed a progressive increase in AKI incidence across tertiles (11.6%, 47.1%, and 91.3%). This model provides an accurate and practical tool for predicting AKI in MOJ patients using routine clinical parameters. Prospective multicenter studies are needed to confirm generalizability.

## 1. Background

Malignant obstructive jaundice (MOJ) is a biliary obstruction syndrome caused by malignant tumors of the biliary system, such as cholangiocarcinoma or pancreatic head cancer. Patients with MOJ are often in a complex clinical state characterized by systemic inflammation, bile stasis, malnutrition, and hepatic and renal dysfunction [1]. Studies have shown that acute kidney injury (AKI) is one of the most common and significant complications in MOJ patients, especially after undergoing percutaneous transhepatic biliary drainage, where renal failure can directly lead to in-hospital mortality [2]. It has been reported that once AKI occurs in MOJ patients, the mortality rate may reach as high as 70–80% [3]. Therefore, the risk of AKI in this population should not be overlooked, and enhanced renal function monitoring and timely intervention are essential.

In the field of AKI prediction, several models have been developed for specific populations such as intensive care unit patients, those with heart failure, sepsis, or acute pancreatitis [4]. In particular, the recent application of machine learning and big data analysis methods has enabled increasingly accurate clinical prediction tools [5]. For example, Demirjian et al. developed a laboratory index-based prediction model in over 60,000 cardiac surgery patients, which demonstrated excellent performance in external validation (area under the curve (AUC) = 0.916), providing a powerful tool for postoperative AKI risk assessment [6]. Additionally, a multicenter study published in 2024 by Su et al. constructed an early prediction nomogram for AKI in sepsis patients using least absolute shrinkage and selection operator (LASSO) and logistic regression. The model achieved high discriminatory performance (AUC > 0.91) in both internal and external validations and outperformed traditional scoring systems such as Sequential Organ Failure Assessment and Acute Physiology and Chronic Health Evaluation II [7].

However, patients with MOJ represent a distinct clinical population with unique risk factors for AKI, such as bile acid accumulation, cholestasis-induced inflammation, and impaired renal hemodynamics. Existing AKI prediction models may not be directly applicable to this group. To date, no predictive tool has been specifically designed for early AKI detection in MOJ patients, highlighting a significant gap in the current literature and clinical practice.

Although recent studies have developed diagnostic models to distinguish benign from malignant causes of obstructive jaundice in large cohorts, such as the machine learning of obstructive jaundice based on common laboratory tests model, these primarily focus on etiology differentiation and do not address the prediction of common acute complications like AKI in MOJ patients [8]. The MOJ population has distinct clinical characteristics and complex etiologies, with kidney injury mechanisms closely related to bilirubin metabolism and biliary pressure changes, making general models poorly applicable to this subgroup [5,9].

Based on this, our study employed propensity score matching (PSM) to establish balanced cohorts of patients with and without AKI, and used LASSO and logistic regression to construct an early risk prediction model for AKI in MOJ patients. The model was evaluated using receiver operating characteristic (ROC) analysis, concordance index (C-index), Brier score, calibration curve, decision curve analysis (DCA), and other metrics. This study fills an important gap in the prediction of AKI in MOJ patients and provides a theoretical and practical tool for the early identification and management of high-risk individuals.

## 2. Patients and methods

### 2.1. Study design and population

This was a single-center retrospective study that included patients diagnosed with MOJ who were hospitalized at Taizhou People’s Hospital between January 2020 and December 2023.

Inclusion criteria were as follows: (1) age ≥18 years; (2) confirmed diagnosis of MOJ; (3) completion of comprehensive laboratory tests within 48 h of admission; (4) a definitive assessment of AKI status based on the Kidney Disease: Improving Global Outcomes (KDIGO) criteria; and (5) complete clinical data.

Exclusion criteria were as follows: (1) history of chronic kidney disease; (2) infection, hemorrhage, or use of nephrotoxic drugs within 7 days prior to admission; (3) current urinary tract obstruction; (4) age <18 years or pregnant/postpartum women; and (5) missing critical data.

**Figure 1.**
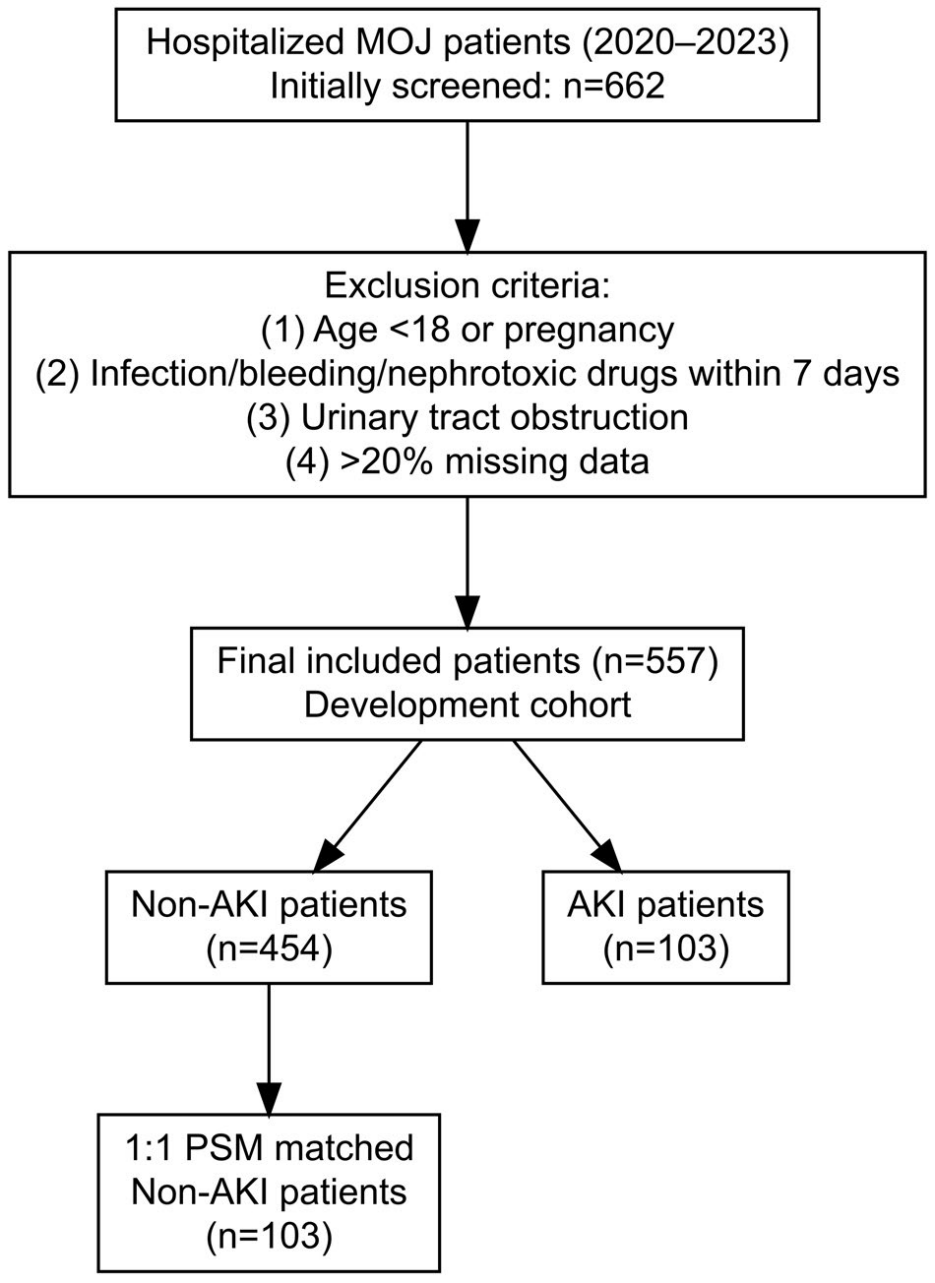
Flowchart of patient inclusion and grouping. A total of 662 malignant obstructive jaundice (MOJ) patients were initially screened, and 557 were included after applying exclusion criteria. Patients were divided into acute kidney injury (AKI) and non-AKI groups, followed by 1:1 propensity score matching (PSM).

A total of 662 patients were initially screened, and 557 met the inclusion criteria. Among them, 103 patients developed AKI, and the remaining 454 did not. To reduce confounding factors, PSM was performed at a 1:1 ratio using the nearest-neighbor method (caliper = 0.02). Matching variables included age, sex, diabetes, hypertension, baseline total bilirubin, albumin, blood urea nitrogen (BUN), and serum creatinine (Scr). After matching, 103 pairs of patients were included for further analysis (Figure 1).

For PSM, baseline data for the AKI group were obtained from laboratory results at admission before the occurrence of AKI, while for the non-AKI group, laboratory results within 48 h of admission were used. Importantly, for the subsequent model construction and analysis, data were collected at different time points from those used for PSM: for AKI patients, variables were obtained within 48 h before AKI onset; for non-AKI patients, variables were obtained within 48 h after admission. This design ensured that PSM balanced baseline characteristics at admission while allowing the analysis dataset to better reflect the patients’ clinical status close to the prediction time point.

### 2.2. Definition of AKI

AKI was defined according to the 2012 KDIGO guidelines as any of the following: an increase in Scr ≥26.5 μmol/L within 48 h, an increase ≥1.5 times the baseline within 7 days, or urine output <0.5 mL/kg/h for more than 6 h.

### 2.3. Data collection and variable definitions

Demographic data (age, sex), comorbidities (diabetes, hypertension), vital signs, and laboratory test results were collected, including complete blood count, biochemical parameters, electrolytes, liver and kidney function, coagulation profiles, and inflammatory markers. Variables with <20% missing data were imputed using multiple imputation by chained equations; those with ≥20% missing data were excluded.

### 2.4. Statistical analysis

All statistical analyses were conducted using R software (version 4.2.2; R Foundation for Statistical Computing, Vienna, Austria). PSM was performed at a 1:1 ratio using the MatchIt package. Matching variables included sex, age, diabetes, hypertension, baseline total bilirubin, albumin, BUN, and Scr. Matching balance was evaluated using standardized mean differences (SMD), with SMD <0.2 indicating good balance.

Continuous variables were presented as mean ± standard deviation (SD) if normally distributed and compared using independent t-tests. Non-normally distributed variables were presented as median (interquartile range) and compared using Mann–Whitney U tests. Categorical variables were expressed as counts (percentages) and compared using the chi-square test.

Missing data were imputed using the mice package. Univariate logistic regression (glm function) was used to screen variables with *p* < 0.1. Variance inflation factor (VIF) was calculated to assess multicollinearity, and variables with VIF > 5 were excluded. The remaining variables were subjected to LASSO regression (glmnet package) to select predictors. The BUN/Scr ratio was considered as a candidate variable given its clinical relevance to volume depletion; however, due to collinearity with its component variables (BUN and Scr), it was not retained in the final candidate set to avoid redundancy and model instability.

A multivariate logistic regression model was then constructed using seven selected variables: neutrophil-to-lymphocyte ratio (NLR), C-reactive protein (CRP), total bilirubin (TBil), uric acid (UA), potassium (K), carbon dioxide combining power (CO_2_CP), and prothrombin time (PT). Predictive performance was assessed using the area under the ROC curve (AUC, pROC), C-index, Brier score, and calibration (Hosmer–Lemeshow test and bootstrap-based calibration with 1,000 repetitions). Internal validation of the final model was performed using bootstrap resampling (*B* = 1,000) to evaluate model optimism and stability. A nomogram was constructed using the rms package, and decision curve analysis (DCA) and clinical impact curves (CIC) were generated using the ggDCA and rmda packages, respectively.

All statistical tests were two-sided, and *p* < 0.05 was considered statistically significant.

## 3. Results

### 3.1. Patient characteristics and propensity score matching

A total of 557 patients with MOJ were included in the study, among whom 103 developed AKI and 454 did not. After 1:1 PSM, 103 matched pairs of AKI and non-AKI patients were identified. Matching variables included sex, age, diabetes, hypertension, baseline total bilirubin, albumin, BUN, and serum creatinine (Table 1). The SMD of all matched variables were <0.2, indicating good matching quality and balanced baseline characteristics between the two groups.

**Table 1.** Comparison of baseline characteristics between acute kidney injury (AKI) and non-AKI groups after propensity score matching.

Variables	AKI	Non-AKI	*t*/*χ*^2^	*p* Value
Sex (male), *n* (%)	66 (64.1%)	59 (57.3%)	0.73	0.392
Age (years)	69 ± 11	70 ± 11	−0.25	0.799
Diabetes, *n* (%)	14 (13.6%)	16 (15.5%)	0.04	0.843
Hypertension, *n* (%)	37 (35.9%)	38 (36.9%)	0.00	1.000
Baseline BUN (mmol/L)	6.26 ± 2.53	5.93 ± 2.39	0.96	0.337
Baseline Scr (μmol/L)	69.0 ± 6.2	67.7 ± 15.5	0.58	0.565
Albumin (g/L)	30.8 ± 4.6	29.4 ± 5.6	1.94	0.053
Total bilirubin (μmol/L)	259.4 ± 152.1	253.6 ± 139.5	0.29	0.776

*Note*. AKI, acute kidney injury; BUN, blood urea nitrogen; Scr, serum creatinine. Data are presented as mean ± SD for normally distributed continuous variables and median (IQR) for non-normally distributed continuous variables; categorical variables are shown as *n* (%). Between-group comparisons used Student’s *t* test or Mann–Whitney U test for continuous variables, and the *χ*² test for categorical variables, as appropriate.

### 3.2. Comparison of baseline characteristics

After matching, several clinical and laboratory indicators significantly differed between the AKI and non-AKI groups (Table 2). Levels of NLR, CRP, UA, TBil, K, and PT were significantly higher in the AKI group compared to the non-AKI group (*p* < 0.05), while CO_2_CP was significantly lower (*p* < 0.05).

**Table 2.** Comparison of baseline clinical characteristics between acute kidney injury (AKI) and non-AKI groups in patients with malignant obstructive jaundice (MOJ).

Variables	AKI	Non-AKI	*t*/*Z*/*χ*²	*p* Value
Sex (male), *n* (%)	66 (64.1%)	59 (57.3%)	0.73	0.392
Age (years)	73 (60, 78)	70 (63, 78)	5284.00	0.962
BMI (kg/m^2^)	21.32 (19.62, 22.99)	21.83 (19.90, 23.59)	5045.00	0.315
Diabetes, *n* (%)	14 (13.6%)	16 (15.5%)	0.04	0.843
Hypertension, *n* (%)	37 (35.9%)	38 (36.9%)	0.00	1.000
SBP (mmHg)	122 ± 2	128 ± 2	2.40	0.017
DBP (mmHg)	74 ± 1	76 ± 1	1.49	0.137
Hemoglobin (g/L)	104 (90, 118)	109 (96, 121)	5915.00	0.154
WBC (×10⁹/L)	9.44 (6.58, 14.22)	6.95 (5.23, 10.17)	3,900.00	0.001
Neutrophil count (×10⁹/L)	7.87 (5.25, 12.26)	5.20 (3.63, 8.05)	3,669.00	<0.001
Lymphocyte count (×10⁹/L)	0.60 (0.38, 0.97)	0.84 (0.57, 1.10)	6,702.00	0.001
Eosinophil count (×10⁹/L)	0.04 (0.01, 0.10)	0.07 (0.02, 0.13)	6,421.00	0.009
Monocyte count (×10⁹/L)	0.58 (0.38, 0.78)	0.50 (0.36, 0.70)	4,468.00	0.051
PLT (×10⁹/L)	146 (96, 200)	199 (134, 243)	6,974.00	<0.001
CRP (mg/L)	51.95 (25.31, 100.69)	12.30 (3.70, 35.58)	1,587.00	<0.001
NLR	11.96 (7.77, 25.02)	5.85 (3.68, 12.38)	3,254.00	<0.001
TBil (μmol/L)	343.5 (213.1, 448.1)	215.3 (150.3, 329.8)	3,516.00	<0.001
DBil (μmol/L)	281.6 (143.2, 351.5)	116.2 (74.7, 168.0)	1,780.00	<0.001
IBil (μmol/L)	146.5 (100.8, 246.5)	99.7 (72.5, 134.1)	2,472.00	<0.001
Albumin (g/L)	29.2 ± 0.5	30.1 ± 0.5	1.19	0.234
ALT (U/L)	61 (39, 122)	96 (56, 206)	6,748.00	<0.001
AST (U/L)	104 (72, 202)	109 (65, 198)	5,258.00	0.914
ALP (U/L)	383 (215, 745)	484 (345, 774)	6,342.00	0.015
LDH (U/L)	280 (217, 400)	266 (201, 355)	3,874.00	0.198
FBG (mmol/L)	5.44 (4.40, 6.59)	5.69 (4.50, 7.49)	5,297.00	0.211
TC (mmol/L)	3.41 (2.35, 4.69)	4.09 (3.38, 5.81)	4,859.00	<0.001
TG (mmol/L)	1.74 (1.23, 2.55)	2.09 (1.32, 2.91)	4,218.00	0.148
HDL-C (mmol/L)	0.56 (0.39, 0.91)	0.66 (0.48, 0.90)	3,986.00	0.110
LDL-C (mmol/L)	2.68 (1.72, 3.32)	3.08 (2.33, 4.27)	4,430.00	0.003
BUN (mmol/L)	13.39 (10.40, 18.84)	5.31 (4.55, 7.42)	632.00	<0.001
Scr (μmol/L)	145.2 (121.9, 196.1)	65.4 (54.7, 79.3)	26.00	<0.001
UA (μmol/L)	325 (248, 410)	225 (175, 284)	2488.00	<0.001
Beta-2 microglobulin (mg/L)	6.59 (4.62, 9.38)	2.30 (2.01, 2.81)	378.00	<0.001
Cystatin C (mg/L)	2.03 (1.58, 2.45)	0.85 (0.76, 1.03)	148.00	<0.001
Potassium (mmol/L)	4.04 (3.54, 4.58)	3.66 (3.27, 3.92)	3,466.00	<0.001
Sodium (mmol/L)	134.0 (130.6, 137.7)	137.1 (134.4, 139.1)	6,958.00	<0.001
Chloride (mmol/L)	99.3 (94.1, 103.6)	101.5 (97.7, 104.5)	6,310.00	0.019
Calcium (mmol/L)	2.12 (2.03, 2.24)	2.16 (2.06, 2.24)	4,754.00	0.191
Magnesium (mmol/L)	0.91 (0.82, 1.04)	0.88 (0.82, 0.92)	3,424.00	0.019
Phosphorus (mmol/L)	1.05 (0.78, 1.33)	0.90 (0.76, 1.08)	3,896.00	0.001
CO₂CP (mmol/L)	21.4 (18.0, 25.2)	25.0 (22.7, 26.9)	6,224.00	<0.001
PT (seconds)	14.0 (12.0, 18.6)	12.1 (11.4, 13.0)	2,703.00	<0.001
INR	1.18 (1.02, 1.48)	1.01 (0.96, 1.07)	2,632.00	<0.001
APTT (seconds)	36.9 (31.8, 43.9)	31.7 (29.1, 35.3)	2,380.00	<0.001
TT (seconds)	18.7 (17.8, 19.7)	18.40 (17.5, 19.4)	3,659.00	0.146
Fibrinogen (g/L)	3.29 (2.52, 4.42)	3.82 (2.98, 4.54)	4,858.00	0.058
D-dimer (mg/L)	4.37 (1.90, 13.96)	1.41 (0.84, 6.16)	2,770.00	<0.001

*Note*. AKI, acute kidney injury; BMI, body mass index; SBP, systolic blood pressure; DBP, diastolic blood pressure; WBC, white blood cell count; PLT, platelet count; CRP, C-reactive protein; NLR, neutrophil-to-lymphocyte ratio; TBil, total bilirubin; DBil, direct bilirubin; IBil, indirect bilirubin; ALT, alanine aminotransferase; AST, aspartate aminotransferase; ALP, alkaline phosphatase; LDH, lactate dehydrogenase; FBG, fasting blood glucose; TC, total cholesterol; TG, triglycerides; HDL-C, high-density lipoprotein cholesterol; LDL-C, low-density lipoprotein cholesterol; BUN, blood urea nitrogen; Scr, serum creatinine; UA, uric acid; CO_2_CP, carbon dioxide combining power; PT: prothrombin time; INR: international normalized ratio; APTT, activated partial thromboplastin time; TT, thrombin time. Data are presented as mean ± SD for normally distributed continuous variables and median (IQR) for non-normally distributed continuous variables; categorical variables are shown as *n* (%). Between-group comparisons used Student’s *t* test or Mann–Whitney U test for continuous variables, and the *χ*² test for categorical variables, as appropriate.

### 3.3. Univariate and multivariate logistic regression analysis

**Figure 2.**
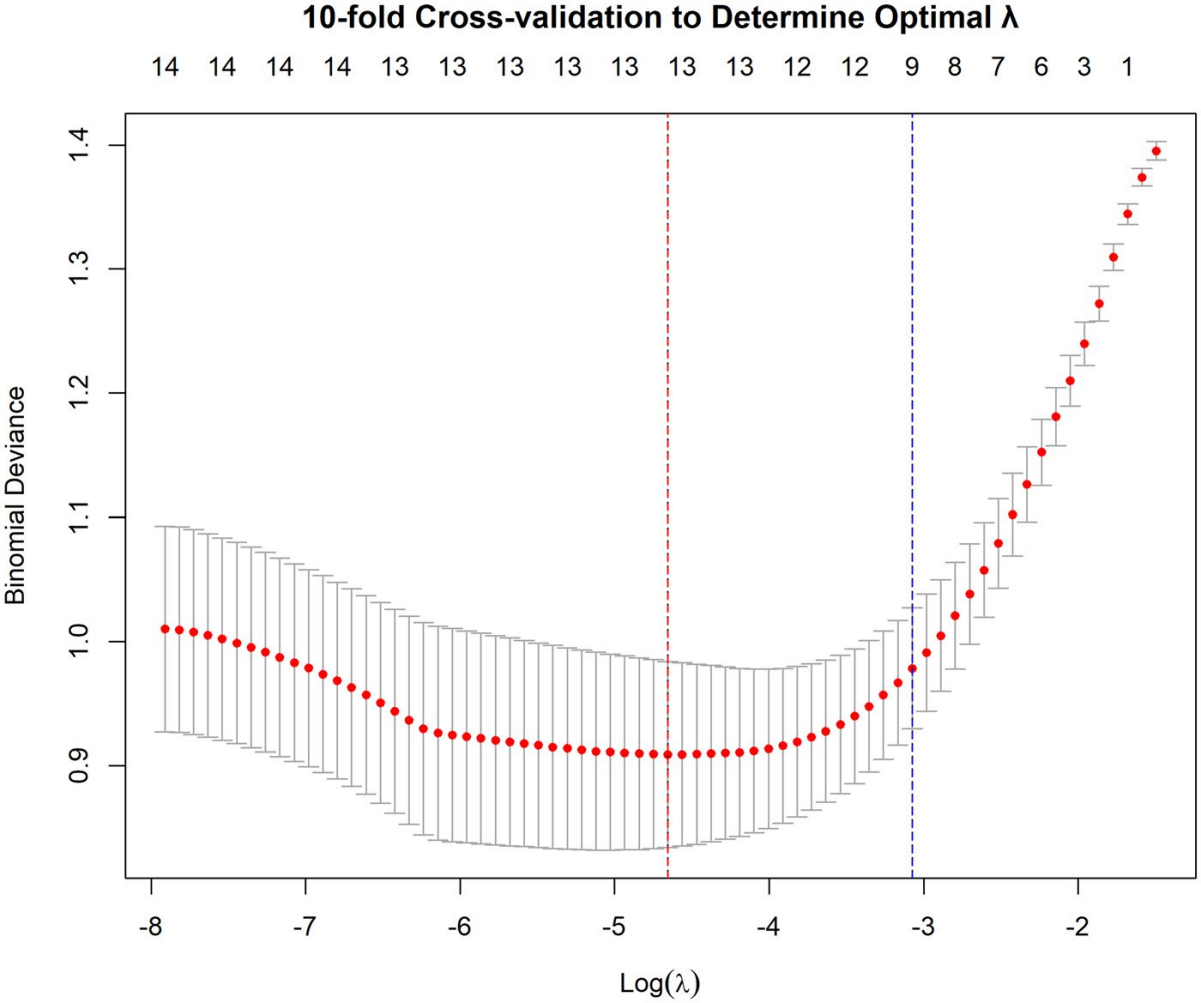
Ten-fold cross-validation for selecting the optimal penalty parameter (*λ*) in least absolute shrinkage and selection operator (LASSO) logistic regression. The red dashed line indicates λmin, and the blue dashed line indicates λ1se.

**Figure 3.**
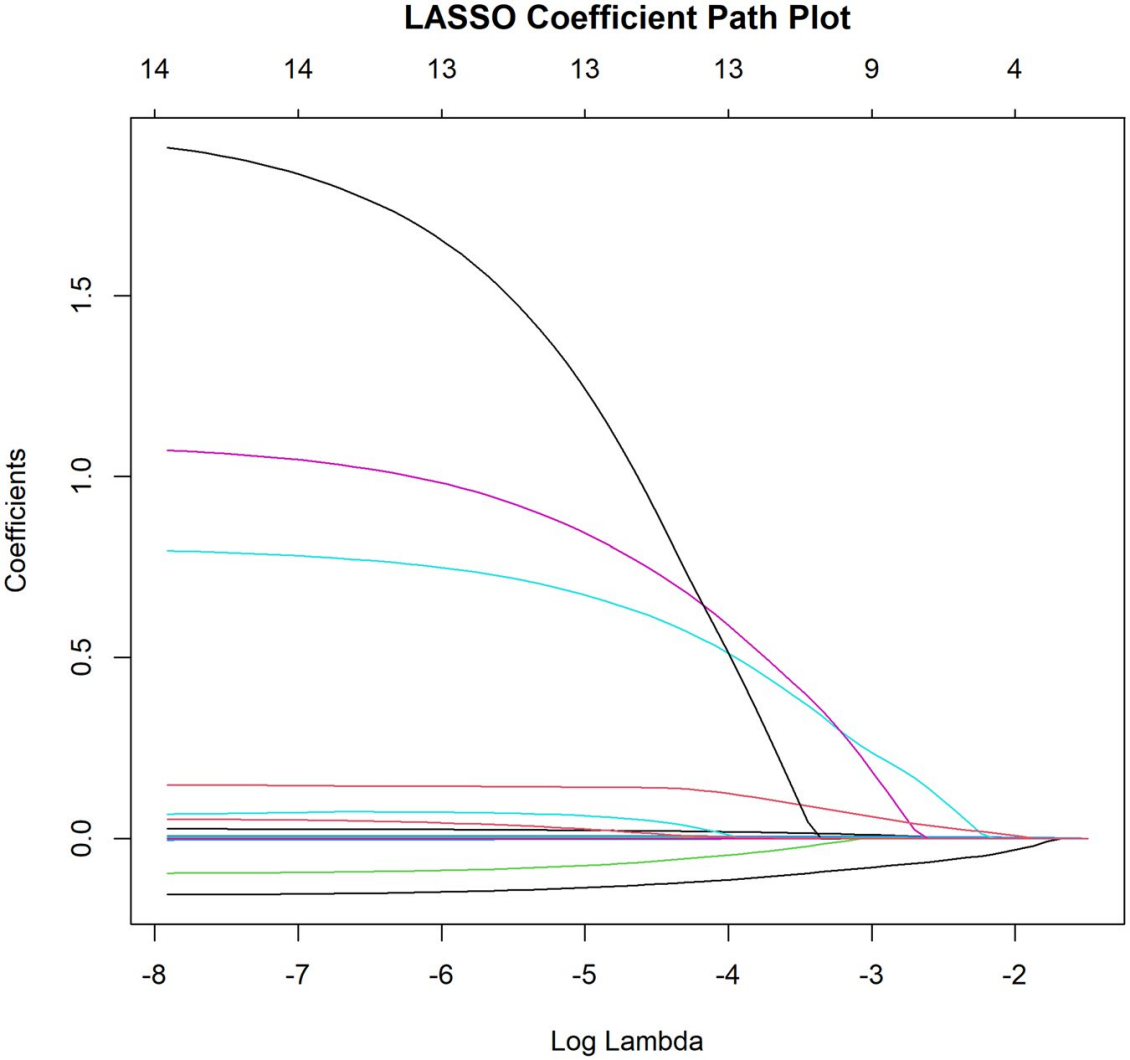
Least absolute shrinkage and selection operator (LASSO) coefficient profiles for the 13 candidate predictors. Coefficients shrink toward zero as regularization strength increases across the log(*λ*) range.

Univariate logistic regression identified 29 variables potentially associated with AKI (*p* < 0.1) (Table 3). Variables with VIF > 5 were excluded to avoid multicollinearity. LASSO regression was then applied for variable selection, yielding 13 candidate predictors (Figures 2 and 3). Based on the LASSO results, univariate P-values, and clinical interpretability, eight variables (NLR, CRP, TBil, UA, K, CO_2_CP, PT, and phosphorus) were included in the multivariate logistic regression model. The final analysis showed that all variables except phosphorus were independent risk factors for AKI (*p* < 0.05) (Table 4).

**Table 3.** Univariate logistic regression analysis for risk factors of acute kidney injury (AKI).

Variable	OR	95% CI	Wald *χ*²	*p* Value
BMI (kg/m^2^)	0.932	0.856–1.011	2.77	0.096
SBP (mmHg)	0.981	0.965–0.997	5.48	0.019
WBC (10^9^/L)	1.087	1.034–1.148	9.90	0.002
Neutrophil count (×10⁹/L)	1.097	1.041–1.161	11.08	<0.001
Lymphocyte count (×10⁹/L)	0.414	0.209–0.772	7.07	0.008
Monocyte count (×10⁹/L)	2.749	1.186–6.869	5.17	0.023
PLT (10^9^/L)	0.995	0.992–0.998	9.39	0.002
CRP (mg/L)	1.010	1.005–1.016	15.44	<0.001
NLR	1.048	1.025–1.076	14.30	<0.001
TBil (μmol/L)	1.004	1.002–1.007	17.75	<0.001
DBil (μmol/L)	1.009	1.006–1.012	31.51	<0.001
IBil (μmol/L)	1.008	1.004–1.012	14.03	<0.001
TC (mmol/L)	0.820	0.717–0.923	9.46	0.002
LDL-C (mmol/L)	0.772	0.649–0.901	9.60	0.002
FBG (mmol/L)	0.883	0.786–0.980	4.95	0.026
BUN (mmol/L)	1.937	1.635–2.382	47.95	<0.001
Scr (μmol/L)	1.321	1.184–1.604	14.01	<0.001
UA (μmol/L)	1.010	1.007–1.014	33.65	<0.001
beta-2 microglobulin (mg/L)	1.111	1.026–1.192	6.25	0.012
Cystatin C (mg/L)	367.316	83.169–2378.194	48.4	<0.001
Potassium (mmol/L)	2.861	1.833–4.686	19.37	<0.001
Sodium (mmol/L)	0.912	0.860–0.962	10.49	0.001
Magnesium (mmol/L)	14.663	2.372–107.710	7.70	0.006
Phosphorus (mmol/L)	5.297	2.290–13.500	13.64	<0.001
CO₂CP (mmol/L)	0.822	0.758–0.884	24.92	<0.001
PT (seconds)	1.378	1.220–1.591	22.48	<0.001
INR	42.321	10.000–233.930	21.66	<0.001
APTT (seconds)	1.109	1.066–1.162	22.46	<0.001
D-dimer (mg/L)	1.000	1.000–1.000	2.83	0.093

*Note*. AKI, acute kidney injury; BMI, body mass index; SBP, systolic blood pressure; WBC, white blood cell count; PLT, platelet count; CRP, C-reactive protein; NLR, neutrophil-to-lymphocyte ratio; TBil, total bilirubin; DBil, direct bilirubin; IBil, indirect bilirubin; TC, total cholesterol; LDL-C, low-density lipoprotein cholesterol; FBG, fasting blood glucose; BUN, blood urea nitrogen; Scr, serum creatinine; UA, uric acid; CO_2_CP, carbon dioxide combining power; PT: prothrombin time; INR: international normalized ratio; APTT, activated partial thromboplastin time; OR, odds ratio; 95% CI, 95% confidence interval. Only show factors with *p* < 0.1.

**Table 4.** Multivariate logistic regression analysis for risk factors of acute kidney injury (AKI).

Variables	*β* value	OR	95%CI	Wald *χ*²	*p* Value
UA (μmol/L)	0.008	1.008	1.004–1.012	15.14	<0.001
PT (seconds)	0.239	1.269	1.109–1.489	10.18	0.001
CO₂CP (mmol/L)	−0.151	0.860	0.773–0.950	8.33	0.004
TBil (μmol/L)	0.003	1.003	1.001–1.006	5.66	0.017
CRP (mg/L)	0.006	1.006	1.001–1.013	5.08	0.024
NLR	0.033	1.034	1.002–1.069	4.24	0.039
Potassium (mmol/L)	0.722	2.058	1.027–4.313	3.94	0.047
Phosphorus (mmol/L)	1.351	3.860	1.030–16.602	3.65	0.056

### 3.4. Model discrimination and performance evaluation

**Figure 4.**
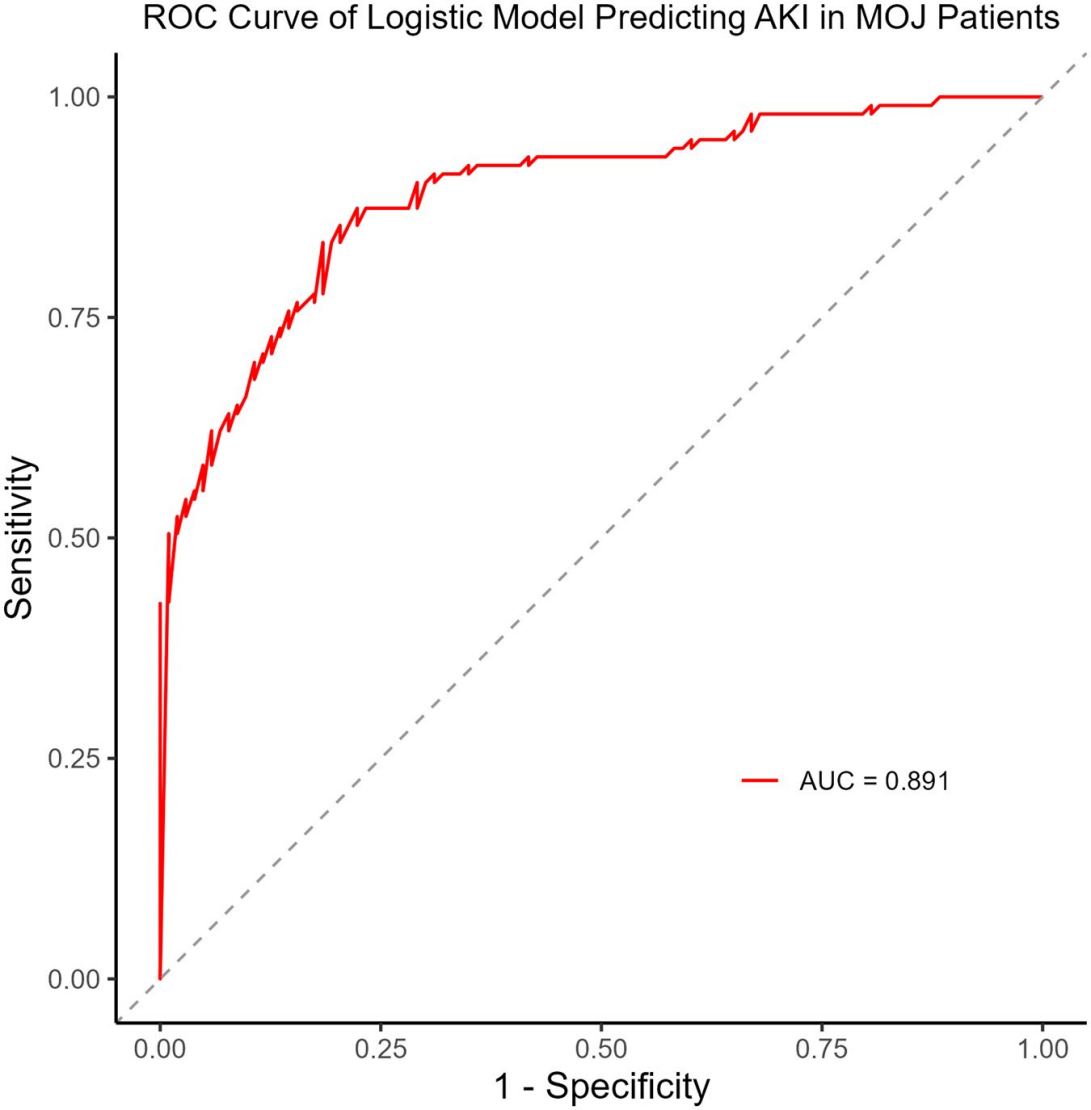
Receiver operating characteristic (ROC) curve of combined predictors for acute kidney injury (AKI) in malignant obstructive jaundice (MOJ) patients. The ROC curve was constructed using a logistic regression model incorporating the following predictors: uric acid (UA), prothrombin time (PT), carbon dioxide combining power (CO_2_CP), total bilirubin (TBil), C-reactive protein (CRP), neutrophil-to-lymphocyte ratio (NLR), and potassium (K). The area under the ROC curve (AUC) was 0.891.

Several models based on different combinations of the seven variables were constructed to evaluate discriminative ability. As the number of variables increased, the model AUC improved accordingly. The full seven-variable model achieved the highest AUC of 0.891, demonstrating excellent discrimination (Figure 4). The model also showed strong performance, with a C-index of 0.890 (95% CI: 0.803–0.977), a Brier score of 0.1317, and a Hosmer–Lemeshow goodness-of-fit test *p* = 0.567. The continuous net reclassification improvement was significant (*p* = 0.00024), although the integrated discrimination improvement was not (*p* = 0.1205) (Table 5).

**Table 5.** Summary of predictive performance of the final logistic regression model.

Metric	Value	Significance
AUC	0.891	Significant
C-index	0.890 (95% CI: 0.803–0.977)	Significant
Brier score	0.1317	Significant
Hosmer–Lemeshow test	*p* = 0.567	Significant
NRI	0.466 (*p* = 0.00024)	Significant
IDI	0.0147 (*p* = 0.1205)	Not significant

*Note*: AUC: area under the receiver operating characteristic curve; C-index: concordance index; NRI: net reclassification improvement; IDI: integrated discrimination improvement.

### 3.5. Model calibration

**Figure 5.**
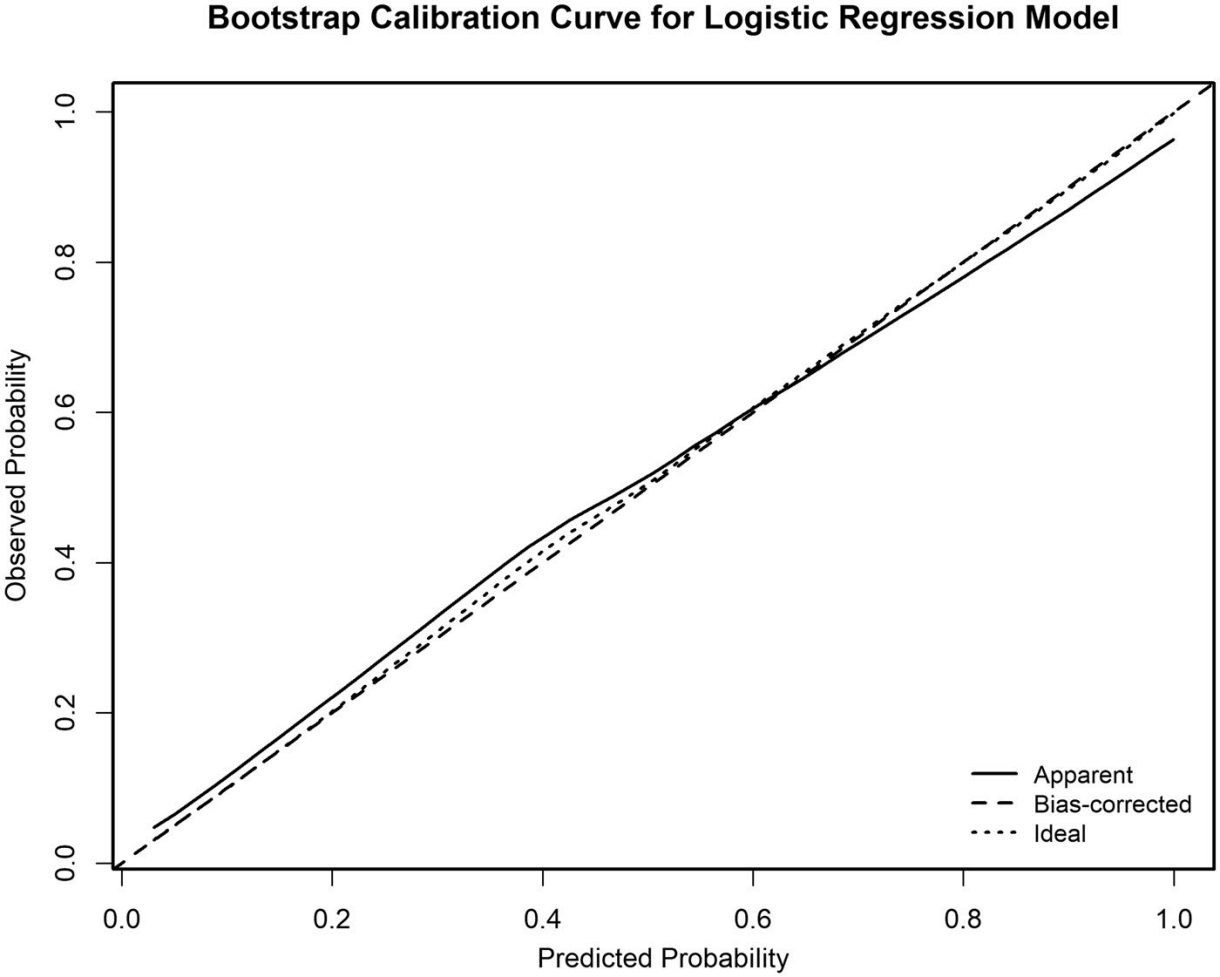
Calibration curve of the logistic regression model for predicting acute kidney injury (AKI) in malignant obstructive jaundice (MOJ) patients. The calibration curve was generated using bootstrap resampling (*B* = 1000) for internal validation of the logistic regression model. The plot shows the apparent predictive curve (gray solid line), the bias-corrected calibration curve (gray dashed line), and the ideal reference line (gray dotted line). The close agreement between predicted and observed probabilities indicates satisfactory calibration performance.

The internally validated calibration plot generated using bootstrap resampling (*B* = 1000) (Figure 5) showed good agreement between predicted probabilities and observed event rates, indicating satisfactory calibration.

### 3.6. Nomogram construction

**Figure 6.**
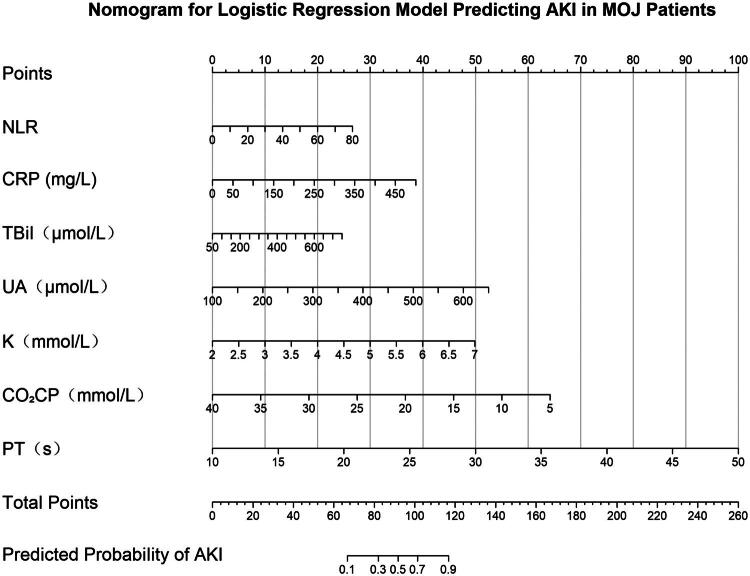
The nomogram was developed based on the logistic regression model for predicting acute kidney injury (AKI) in malignant obstructive jaundice (MOJ) patients, incorporating the predictors defined in Figure 4 (NLR, CRP, TBil, UA, K, CO_2_CP, and PT). Each variable corresponds to a specific score on the nomogram, with the total score indicating the predicted probability of AKI. NLR, neutrophil-to-lymphocyte ratio; CRP, C-reactive protein; TBil, total bilirubin; UA, uric acid; K: potassium; CO_2_CP, carbon dioxide combining power; PT: prothrombin time.

A nomogram was developed based on the final multivariate model (Figure 6), visually demonstrating the contribution of each predictor to AKI risk and enabling individualized clinical risk assessment.

### 3.7. Decision curve analysis

**Figure 7.**
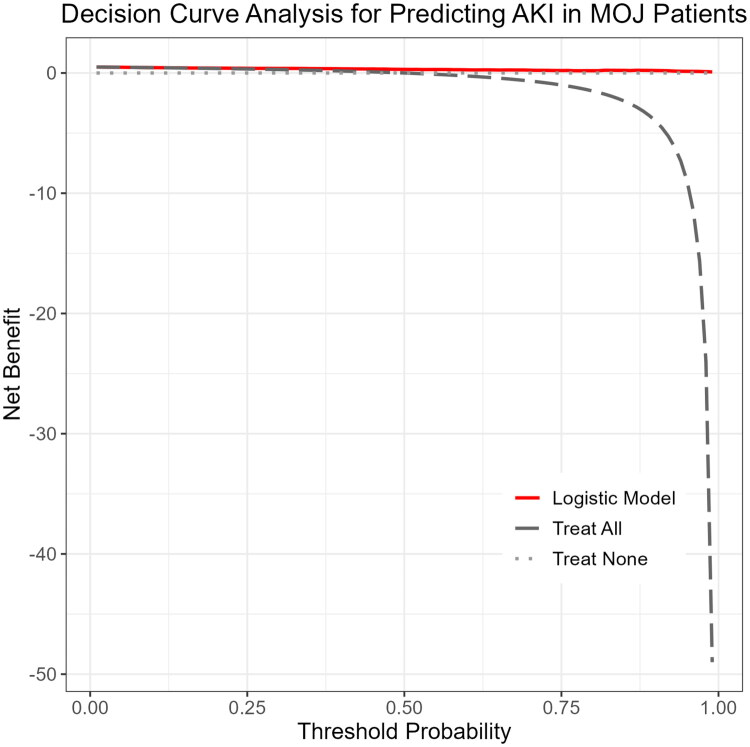
Decision curve analysis (DCA) for predicting acute kidney injury (AKI) in malignant obstructive jaundice (MOJ) patients. The DCA was used to evaluate the clinical utility of the logistic regression model for predicting AKI in patients with MOJ. The red line represents the net benefit of using the prediction model at different threshold probabilities, while the gray dashed and dotted lines represent the strategies of treating all or no patients, respectively. The model demonstrates superior net benefit across a wide range of threshold probabilities, indicating its potential clinical applicability.

The DCA results showed that, within a threshold probability range of 0.1 to 0.8, the logistic model yielded greater net clinical benefit compared to the treat-all or treat-none strategies (Figure 7), supporting its potential clinical applicability.

### 3.8. Risk stratification analysis

**Figure 8.**
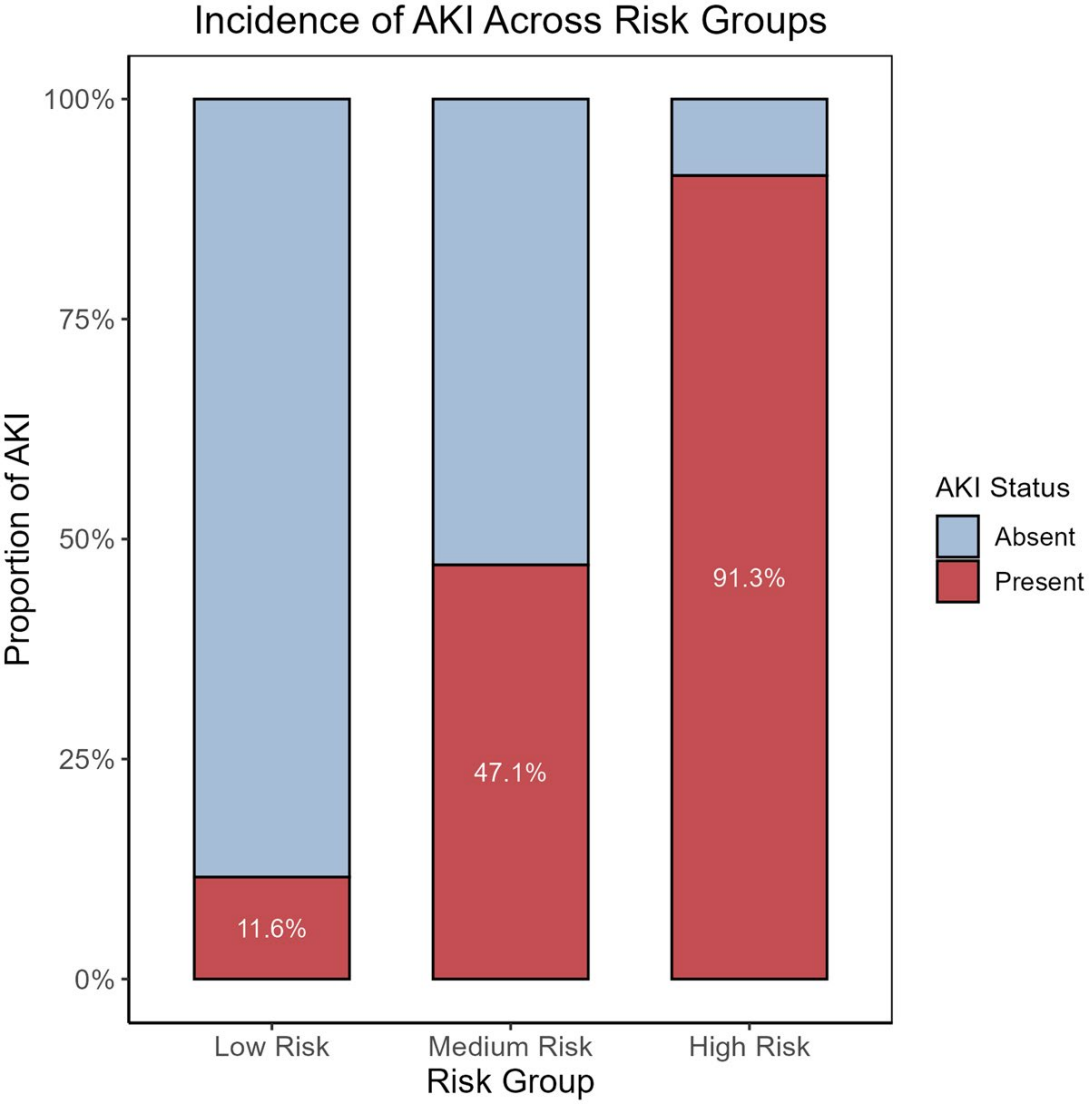
Incidence of acute kidney injury (AKI) across risk strata based on predicted probability. Patients were stratified into low-, medium-, and high-risk groups according to predicted probabilities from the logistic regression model. The bar chart illustrates the proportion of AKI in each group, with incidence rates of 11.6%, 47.1%, and 91.3%, respectively. The difference in AKI incidence among the groups was statistically significant (*χ*^2^ = 88.03, *p* < 0.001).

Based on the nomogram-predicted probabilities, patients were stratified into low-, medium-, and high-risk groups using tertiles. The observed AKI incidence in these groups was 11.6%, 47.1%, and 91.3%, respectively (Figure 8*;* Table 6).

**Table 6.** Incidence of acute kidney injury (AKI) across risk groups and statistical comparison among groups.

Risk group	Total (*n*)	AKI (*n*)	AKI Incidence	*χ*²	*p* Value
Low Risk	69	8	11.6		
Medium Risk	68	32	47.1		
High Risk	69	63	91.3	88.03	<0.001

*Note*. AKI, acute kidney injury. The difference in AKI incidence among the three risk groups was statistically significant (*χ*^2^ = 88.03, *p* < 0.001).

**Figure 9.**
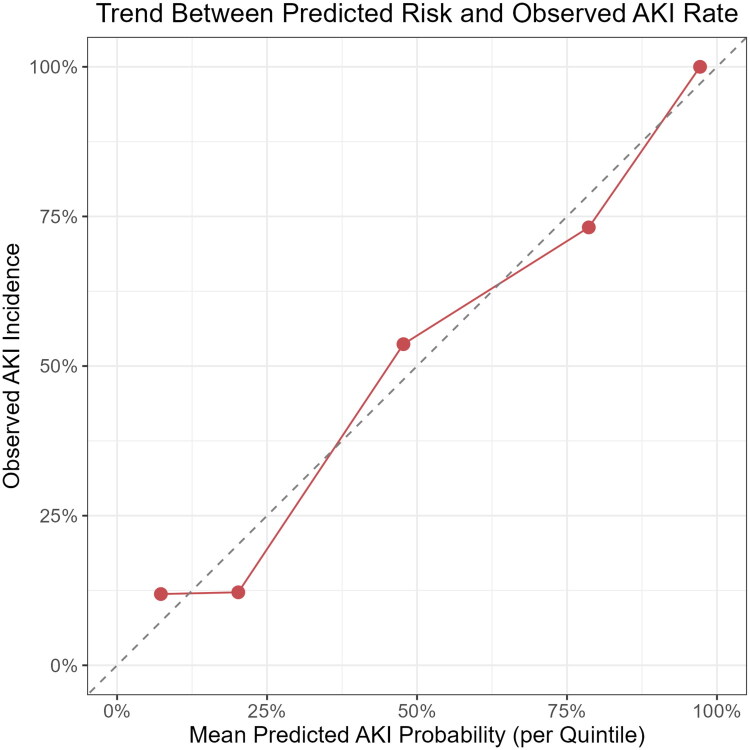
Trend of acute kidney injury (AKI) incidence across risk groups stratified by predicted probability. Patients were classified into tertiles based on predicted risk from the logistic regression model. A progressive increase in AKI incidence was observed across the risk groups, indicating good discrimination and calibration performance. The trend was statistically significant (*p* < 0.001).

Furthermore, a significant upward trend in AKI incidence was observed across the risk strata (χ^2^ = 88.03, *p* < 0.001), demonstrating good risk discrimination of the model (Figure 9).

These risk strata may help guide clinical decision-making by identifying high-risk patients who may benefit from intensified monitoring or early nephrology consultation.

### 3.9. Clinical impact curve

**Figure 10.**
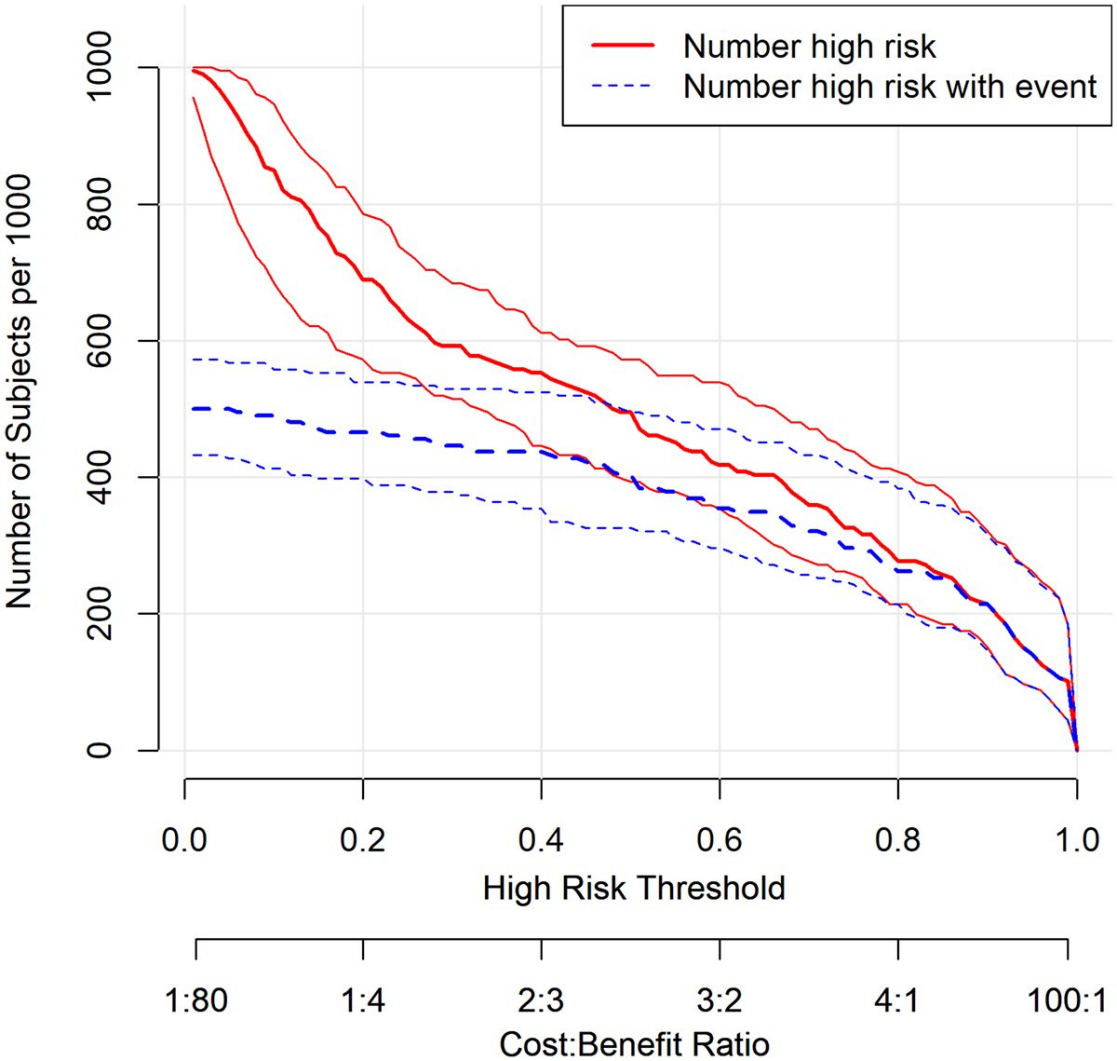
Clinical impact curve (CIC) of the logistic regression model for predicting acute kidney injury (AKI) in patients with malignant obstructive jaundice (MOJ). The red curve represents the number of patients classified as high risk at each threshold, while the blue dashed curve represents the number of true positive cases (patients with AKI) among them. The closer the two curves are, the better the model’s clinical utility.

The CIC analysis (Figure 10) demonstrated close agreement between the number of patients identified as high risk and the actual number of AKI events across different risk thresholds, further supporting the model’s clinical discriminative power and generalizability.

## 4. Discussion

This study found that the incidence of AKI among patients with MOJ was 18.5% (103/557), which is consistent with previously reported rates of AKI in obstructive jaundice patients [3,10]. By applying rigorous PSM, baseline characteristics between the AKI and non-AKI groups were well balanced, thus providing a reliable foundation for model development.

The mechanisms underlying AKI in obstructive jaundice remain incompletely understood.

Current evidence suggests that bile acid accumulation may cause renal tubular injury through oxidative stress and inflammatory responses, ultimately leading to renal dysfunction [11–13]. In addition, inadequate fluid replacement following external biliary drainage in some MOJ patients may result in volume depletion-induced AKI [14]. Although the BUN/Scr ratio is a widely used bedside indicator of prerenal azotemia and volume depletion—often rising disproportionately when intravascular volume is compromised—it was not retained in our model due to collinearity with BUN and serum creatinine. Nevertheless, its clinical utility for identifying potential hypovolemia remains important for early management in MOJ patients and warrants further evaluation in future studies. In this study, TBil was identified as an independent predictor of AKI, suggesting that it is not only an important laboratory indicator for hepatobiliary diseases but can also indirectly reflect the severity of MOJ and may be involved in the development of AKI through pathophysiological processes associated with cholestasis. Furthermore, inflammatory markers such as NLR and CRP were identified as independent risk factors for AKI, consistent with previous findings [15,16]. Elevated NLR reflects systemic inflammation and may participate in AKI pathogenesis *via* inflammation-mediated injury [17]. CRP, as an acute-phase protein, is indicative of ongoing inflammation and has been repeatedly associated with impaired renal function [16,18].

Notably, this study is the first to identify elevated UA as an independent predictor of AKI in MOJ patients. Previous research indicates that high UA levels can induce renal vasoconstriction and inflammation, reducing renal perfusion and accelerating the onset and progression of AKI [19,20]. Elevated UA also reflects increased oxidative stress, which may further exacerbate the vulnerability of renal function in MOJ patients [21,22].

Electrolyte abnormalities were also included in the model. Elevated serum K and decreased CO_2_CP were both found to be independent predictors of AKI. Hyperkalemia may indicate impaired tubular function, epithelial injury, or decreased glomerular filtration rate [23]. A prospective study by Chávez-Íñiguez et al. confirmed that persistent hyperkalemia in AKI patients significantly increased 10-day mortality and the need for renal replacement therapy, underscoring its prognostic significance [24]. Reduced CO_2_CP suggests possible metabolic acidosis, which is closely associated with renal impairment. This further supports its value as a predictive marker for AKI in MOJ patients [25]. Persistent acidosis has also been linked to elevated inflammation and mortality in critically ill AKI patients [26].

In addition, prolonged PT was identified as an independent risk factor for AKI in MOJ patients. PT prolongation often reflects coagulation dysfunction and impaired hepatic synthetic function, indicating that coagulation imbalance may contribute to AKI pathogenesis. This may result from liver insufficiency and decreased synthesis of coagulation factors due to bile stasis [27]. Notably, a recent study by Zhang et al. using an AutoML algorithm also recognized PT as a key predictor of AKI, further validating its clinical relevance [28].

Although several AKI prediction models based on machine learning and logistic regression have been proposed in recent years [29,30], few models specifically target the MOJ population. In this study, we developed an AKI prediction model for MOJ patients using LASSO and multivariate logistic regression, incorporating seven readily available clinical indicators. The model demonstrated high predictive performance (AUC = 0.891). Compared to conventional tools such as Sequential Organ Failure Assessment and Acute Physiology and Chronic Health Evaluation II scores, our model showed superior accuracy and clinical applicability.

The clinical utility of the model was further evaluated through DCA and CIC. The results indicated favorable net benefit and risk discrimination across a wide threshold probability range (0.1–0.8), supporting the model’s value in early identification of high-risk patients.

In summary, we developed and validated an early risk prediction model for AKI in MOJ patients. The model exhibited robust discrimination and strong clinical applicability, providing a scientific basis and practical tool for timely identification and intervention in high-risk individuals.

## 5. Limitations

This single-center retrospective study cannot establish causal relationships, and the findings require confirmation in prospective multicenter studies. Only baseline indicators within 48 h of admission were included, without assessing dynamic changes during hospitalization, and AKI diagnosis was based solely on serum creatinine, potentially underestimating its incidence. Although PSM and LASSO regression were used, some confounders may have been missed. Internal validation was performed using bootstrap resampling (*B* = 1,000) and multiple performance metrics, but the lack of external validation limits generalizability, which should be addressed in future multicenter prospective studies. Despite these limitations, the model showed good discrimination and calibration, offering a practical tool for early AKI identification in MOJ patients.

## 6. Conclusion

This study developed and validated a logistic regression model to predict the occurrence of AKI in hospitalized patients with MOJ, based on seven variables (NLR, CRP, UA, TBil, K, CO_2_CP, and PT) selected after PSM. The model demonstrated good discriminative performance (AUC = 0.891), calibration, and clinical applicability. A nomogram based on the model was constructed to facilitate individualized risk prediction.

The findings provide a reliable basis for the early identification and clinical intervention of AKI in MOJ patients and have considerable potential for clinical translation. However, as this was a single-center retrospective study, further validation in prospective, multicenter cohorts is necessary to confirm the model’s robustness and generalizability.
